# Dynamic Transcriptome Profiling Reveals Key Regulatory Networks Underlying Curd Development in Cauliflower (*Brassica oleracea* L. *botrytis*)

**DOI:** 10.3390/ijms27031308

**Published:** 2026-01-28

**Authors:** Shuting Qiao, Xiaoguang Sheng, Mengfei Song, Huifang Yu, Jiansheng Wang, Yusen Shen, Sifan Du, Jiaojiao Li, Liang Sun, Honghui Gu

**Affiliations:** 1 Department of Vegetable Science, College of Horticulture, China Agricultural University, Beijing 100193, China; qst20210306@126.com; 2 Institute of Vegetables, Zhejiang Academy of Agricultural Sciences, Hangzhou 310021, China; xguang@zaas.ac.cn (X.S.); smf993@163.com (M.S.); yuhf@zaas.ac.cn (H.Y.); wangjs@zaas.ac.cn (J.W.); shenyusen@zaas.ac.cn (Y.S.); dsf0818@163.com (S.D.); 13479339212@163.com (J.L.); 3 Department of Life Sciences, China Jiliang University, Hangzhou 310018, China; 4 Department of Horticultural Science, Zhejiang Agriculture and Forestry University, Hangzhou 311300, China

**Keywords:** cauliflower, curd, peduncle, transcriptome, differentially expressed genes

## Abstract

Cauliflower (*Brassica oleracea* var. *botrytis*) curd formation is a highly complex developmental process governed by tightly coordinated genetic and physiological regulation. Here, we performed transcriptome sequencing of curd and peduncle tissues across multiple developmental stages, generating 171.52 Gb of high-quality data. Genes associated with photosynthesis and glucosinolate biosynthesis were strongly upregulated in the shoot apical meristem (SAM), highlighting substantial metabolic investment during the pre-initiation phase of curd morphogenesis. Key floral transition regulators, particularly *AP2* and MADS-box transcription factors, were activated to drive the vegetative-to-reproductive switch and initiate curd primordia, ultimately giving rise to the arrested inflorescence architecture characteristic of cauliflower. Furthermore, hormone signaling pathways—including auxin (AUX), jasmonic acid (JA), and brassinosteroid (BR)—showed marked activation during SAM proliferation and peduncle elongation, underscoring their crucial roles in structural patterning. Collectively, our findings delineate an integrated regulatory network that links metabolic activity, hormone signaling, and developmental programs, providing novel molecular insights into curd formation and identifying potential breeding targets for the genetic improvement of Brassicaceae crops.

## 1. Introduction

Cauliflower (*Brassica oleracea* var. *botrytis*, 2n = 2x = 18) is a diploid *Brassica* species domesticated from the European wild cabbage (*Brassica oleracea* var. *oleracea*) [1]. This agriculturally important crop is characterized by its edible curd, a quasi-hemispherical morphological structure, resulting from the proliferation and arrested differentiation of apical inflorescence meristems (AIM). The curd is nutritionally valuable, containing high levels of digestible carbohydrates, plant proteins, and essential micronutrients including ascorbic acid and dietary minerals [2]. Notably, cauliflower also contains abundant glucosinolates and their hydrolysis products, which possess well-documented anticancer properties, contributing to its widespread consumer appeal [3,4].

The cauliflower curd functions as both a reproductive structure and a commercially important organ, with its morphological quality directly determining the crop’s market value. A mature curd typically exhibits a near-hemispherical architecture, comprising a proliferative AIM at the surface and numerous branched peduncles forming its basal framework. To date, studies have primarily focused on the regulatory mechanisms governing AIM development and floral transition. Several genes implicated in curd development have been identified, including those associated with AIMs (*TERMINAL FLOWER*, etc.) and floral meristems (*LEAFY*, *APETALA1*, *CAULIFLOWER*, etc.), as well as components of the complex regulatory networks integrating these genes with hormone signaling pathways [5,6]. One notable study constructed the SALT (Self-Similar Asymptotic Limited Transformation) gene regulatory network and coupled it with dynamic morphological modeling, providing mechanistic insights into how cauliflower’s fractal-like curd structure emerges from meristem development programs governed by specific spatiotemporal parameters [6]. Structural variation in the promoter region of the *BobCAL* gene during floral meristem arrest has also been proposed as a potential cause underlying curd formation in cauliflower [7]. Further genetic dissection using linkage mapping based on the Kosambi function and composite interval mapping algorithms has led to the identification of 20 quantitative trait loci (QTLs) associated with curd-related structural traits, including basal diameter, stalk length, stalk angle, and curd solidity [8]. Additionally, the recent release of a high-quality reference genome assembly (C-8, V2) enabled genome-wide association studies (GWAS) that uncovered nine loci significantly associated with curd morphology and developmental traits. Among them, a zinc finger protein gene (*BOB06G135460*) was shown to positively regulate stem height in cauliflower [9].

As cauliflower curds develop, the associated peduncles retain a green coloration and fleshy texture, reflecting their sustained photosynthetic capacity. Subsequently, under favorable environmental conditions, such as appropriate temperature and photoperiod, the curd regains the capacity to differentiate into floral meristematic tissue. This transition is accompanied by the rapid elongation of peduncle stalk, culminating in floral initiation and bolting [10,11]. A genome-wide association study (GWAS) focusing on the length of the outermost branch (LOB) and the second branch (LSB) of the cauliflower curd identified 64 loci significantly associated with branch elongation and developmental architecture [12]. Plant hormones also play a pivotal role in regulating peduncle development [13,14].

Overall, compared to the long history and achievements in cauliflower breeding, studies on its molecular biology have lagged behind. Although previous studies have identified candidate genes or pathways involved in regulating curd development, the key genes and molecular regulatory networks underlying this process remain poorly understood. This knowledge gap has hindered the development of high-quality germplasm and the breeding of improved cauliflower varieties. In this study, we performed transcriptome analyses on three distinct cauliflower tissues—shoot apical meristem (SAM), AIM, and peduncle—to identify key genes and regulatory pathways involved in curd development and peduncle elongation. The findings provide a theoretical foundation for understanding the molecular basis of cauliflower reproductive development and offer valuable insights for future molecular breeding efforts.

## 2. Results

### 2.1. Statistics and Quality Analysis of Transcriptome Sequencing Data

In this study, the plant materials used for transcriptome analysis are summarized as follows. SAM samples were collected from the apex of 25-day-old seedlings at the four-true-leaf stage. Curd samples were collected at four developmental stages: the curd initiation stage (CI), curd thickening stage (CT), curd maturation stage (CM), and curd bolting stage (CB). In addition, peduncle samples were collected at three stages corresponding to curd development: curd thickening stage (CT-P1, peduncle expansion stage), curd maturation stage (CM-P2, peduncle maturation stage), and curd bolting stage (CB-P3, peduncle elongation stage). For each sample type, three biological replicates were collected and immediately frozen in liquid nitrogen for subsequent RNA-Seq analysis.

To obtain transcriptome data from different developmental stages of cauliflower, we performed RNA sequencing on 24 samples representing various curd and peduncle developmental stages (Table 1 and Table S1). A total of 171.52 Gb of high-quality clean data was generated. Each sample yielded over 6.06 Gb of clean data, with clean read ratios ranging from 96.41% to 98.23%. Following quality control, the percentage of bases with Q30 (error rate ≤ 0.1%) exceeded 89.56%, and bases with Q20 (error rate ≤ 1%) exceeded 96.32%. Clean reads were aligned to the cauliflower Korse reference genome [7] using HISAT2 software v2.2.1, achieving mapping rates between 92.96% and 95.45%, with more than 88.21% of reads uniquely mapped (Table 1). These metrics indicate high sequencing quality and reliability, supporting their suitability for downstream analyses.

**Figure 1:**
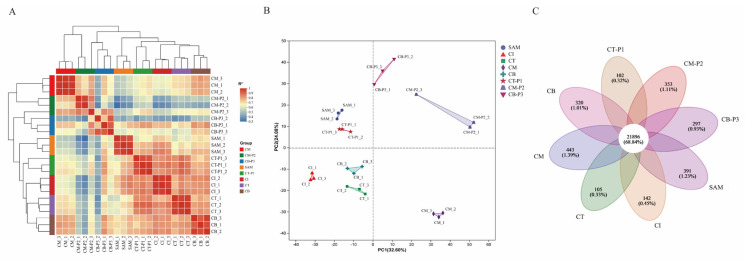
Correlation analysis between all 24 samples. (**A**) Pearson correlation coefficients of samples. (**B**) PCA analysis of samples. (**C**) Venn diagram showing the number of commonly or uniquely expressed genes.

A total of 46,611 expressed genes were identified in this analysis, comprising 43,007 known genes and 3604 novel genes (Table S1). Pearson correlation analysis of the 24 datasets, visualized as a heatmap, revealed high reproducibility (coefficients near 1) among three biological replicates (Figure 1A). Principal component analysis (PCA) was employed to reduce data dimensionality and to explore relationships and variance among samples. The clustering of samples indicates that the differences between groups are significant, while the three replicates within each group are consistently clustered together, suggesting minimal variation (Figure 1B). Additionally, 21,896 genes, accounting for 68.84% of the total detected genes, were commonly expressed across curd and peduncle samples at all developmental stages (Figure 1C).

### 2.2. Identification of Differentially Expressed Genes (DEGs) Between SAM and Three Stages of Curd Development

**Figure 2:**
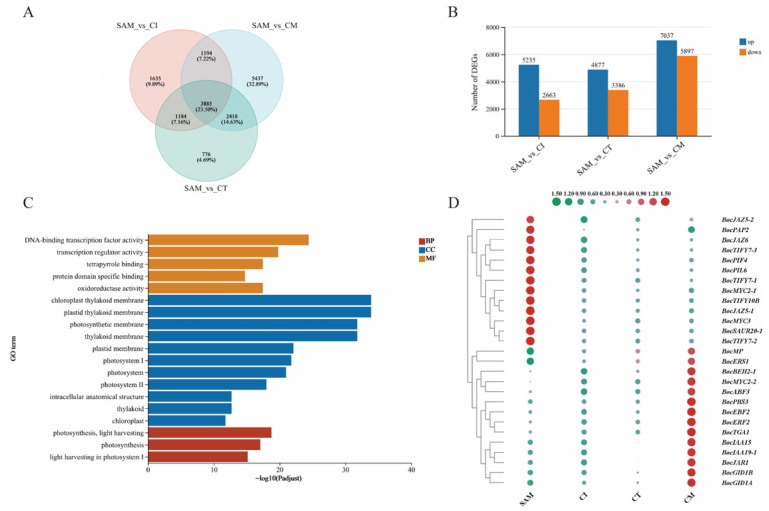
DEGs identified in three pairwise comparisons (SAM vs. CI, SAM vs. CT, and SAM vs. CM). (**A**) Venn diagram of DEGs commonly or specifically expressed between SAM and three stages of curd development. (**B**) Number of expressed genes between SAM and curd developmental stages. (**C**) GO functional enrichment of DEGs between SAM and curd developmental stages. (**D**) Phytohormone signaling DEGs: SAM vs. curd development.

In order to detect potential regulatory genes for the transition from nutritional growth stage of SAM to reproductive growth stages of curd formation, gene transcription levels were compared between SAM and AIM at three different developmental stages (CI, CT and CM). The generated transcriptome data were used to screen DEGs according to the following criteria, Padjust < 0.05 and |log2FC| ≥ 1. A Venn diagram analysis revealed the numbers of DEGs identified in each comparison group (Figure 2A). Specifically, in the comparison between the SAM and CI group, there were 5235 DEGs upregulated and 2663 DEGs downregulated. In the comparison between the SAM and CT group, there were 4877 DEGs upregulated and 3386 DEGs downregulated. The largest number of DEGs were identified in the SAM_vs_CM group, with 7037 genes showing upregulated expression and 5897 genes—downregulated expression (Figure 2B).

A total of 3885 DEGs were found to be in the overlapping region between the SAM and AIM at three different developmental stages (CI, CT, CM) (Figure 2A). Therefore, the possible genetic regulatory networks and pathways enriched by these 3885 overlapping DEGs were explored by GO and KEGG enrichment analysis. The GO enrichment analysis performed on the 3885 overlapping DEGs showed that GO terms were significantly enriched in the pathways related to photosynthesis and transcription regulator activity, such as photosynthesis light harvesting (GO:0009765), photosystem I/Ⅱ (GO:0009522), chloroplast thylakoid membrane (GO:0009535) and DNA-binding transcription factor activity (GO:0003700 and GO:0140110) (Figure 2C). The KEGG pathway enrichment analysis indicated that the plant hormone pathway was most significantly enriched, and the photosynthesis (map00195) and carbon fixation in photosynthetic organisms (map00710) were also enriched. Interestingly, pathways containing carbon and sulfur metabolites are also enriched, such as Starch and sucrose metabolism (map00500), Glucosinolate biosynthesis (map00966) and Sulfur metabolism (map00920) (Figure S1).

Integrated transcriptomic analysis revealed that 47 photosynthesis-related genes—including chlorophyll A/B-binding proteins (CAB), Photosystem I/II subunits (PSI/PSII), and aminoacyl-tRNA synthetases (CAAD)—were significantly upregulated in the SAM, suggesting enhanced light harvesting and chloroplast activity during SAM establishment. Concurrently, 13 phytohormone-associated DEGs, notably bHLH-class transcription factors (TFs) of *BolK_7g30730*, *BolK_5g37040* and *BolK_6g43880* in auxin/jasmonate pathways, were upregulated, implicating their roles in SAM-to-curd transition. Additionally, all DEGs linked to glucosinolate biosynthesis (e.g., cytochrome P450s, glycosyltransferases, sulfotransferases) showed marked SAM upregulation, identifying the SAM as a primary site for glucosinolate production (Tables S2–S4, Figure 2D).

**Figure 3:**
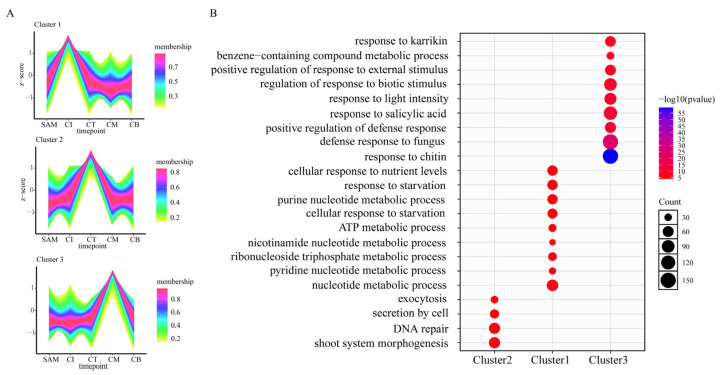
Stage-specific expression profiles and associated GO terms during curd development. (**A**) Mfuzz clustering identified distinct gene expression patterns across curd developmental stages. (**B**) GO enrichment analysis of selected clusters highlights stage-specific biological processes. Circle size corresponds to the number of genes, and color represents the *p* value.

However, among these 3885 DEGs, we did not identify any gene clusters showing consistent upregulation throughout curd development. Therefore, we performed Mfuzz [15] clustering analysis coupled with ggplot2 [16] visualization, which revealed distinct stage-specific expression patterns across curd different developmental phases (Figure 3A). During the CI stage representing the initial phase of curd development, the enrichment of pathways indicates a metabolic shift toward energy production and biosynthesis, likely supporting cell growth and proliferation. These pathways involved cellular response to nutrient levels (GO:0031669), purine nucleotide metabolic process (GO:0006163), ATP metabolic process (GO:0046034), nucleotide metabolic process (GO:0046496), reflecting a high demand for energy and metabolic precursors. Several key genes, such as *BolK_1g01560* (PEPCK_ATP), *BolK_1g56640* (PLDc), *BolK_1g46260* (Metallophos), *BolK_2g10910* (SPX), and *BolK_2g30690* (Put_Phosphatase), were significantly upregulated during this stage, further supporting the activation of these processes. At the CT stage (rapid curd proliferation phase), GO enrichment reveals coordinated developmental processes: shoot morphogenesis (GO:0010016) ensures proper meristem patterning, DNA repair (GO:0006281) maintains genomic stability during rapid division, and cellular secretion (GO:0032940) facilitates cell wall remodeling and intercellular communication. During the CM stage, maturing curds show increased susceptibility to pathogens and mechanical damage. Enriched GO terms reveal heightened sensitivity to diverse signals: karrikin (GO:0080167) for growth regulation, salicylic acid (GO:0009751) and chitin (GO:0010200) for pathogen defense, light intensity (GO:0009642) for environmental adaptation, and benzene compounds (GO:0042537) for stress response (Figure 3B).

### 2.3. DEGs Between CB (Start of Flowering) and Three Stages of Curd Development (CI, CT and CM)

**Figure 4:**
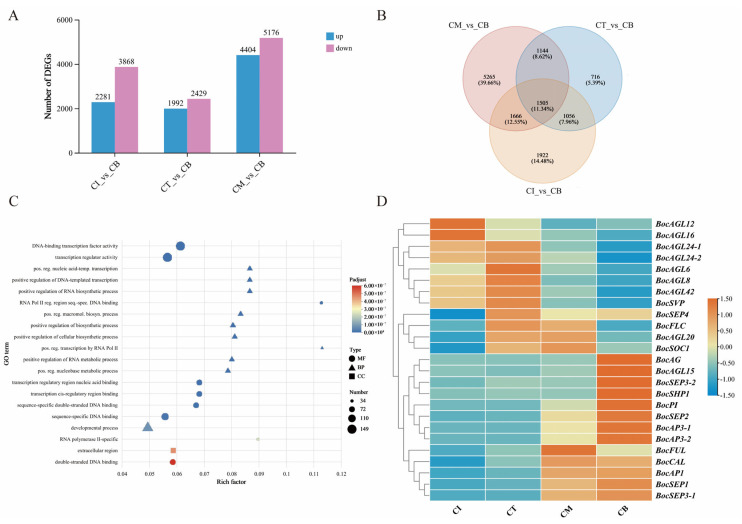
DEGs identified in three pairwise comparisons (CB vs. CI, CB vs. CT, and CB vs. CM). (**A**) Number of expressed CB and three stages of curd development. (**B**) Venn diagram of DEGs commonly or specifically expressed between CB and curd development. (**C**) GO functional enrichment of DEGs between CB and curd development. (**D**) MADS-box transcription factor DEGs: SAM vs. curd development.

The curd development includes three sequential stages of CI, CT and CM, during which the peduncle undergoes slow elongation while the AIM were fully developed. When the curd development is in the CB stage, the peduncle begins to elongate rapidly, and the AIM transforms into floral meristems (FM), marking the beginning of flowering. The FM of CB was compared to the AIM of CI, CT and CM, respectively. Specifically, in the comparison between the CB and CI group, there were 2281 DEGs upregulated and 3868 DEGs downregulated. In the comparison between the CB and CT group, there were 1992 DEGs upregulated and 2429 DEGs downregulated. The largest number of DEGs were identified in the CB and CM group, with 4404 genes showing upregulated expression and 5176 genes—downregulated expression (Figure 4A). A total of 1505 overlapping DEGs, constituting 11.34% of the total detected genes, were identified in the comparison of CB to CI, CT and CM (Figure 4B). Both GO and KEGG enrichment analyses identified significant enrichment in DNA-binding transcription factor activity and transcription regulator activity pathways, suggesting their crucial regulatory roles in cauliflower’s flowering transition process (Figure 4C and Figure S2 ).

Our comparative transcriptomic analysis revealed 146 TF-related DEGs, with seven predominant TF families emerging: MADS-box, AP2/ERF, MYB, C2H2 zinc finger, TCP, WRKY, and bZIP. Within the MADS-box gene family, 12 DEGs containing reported flowering-promoting factors (e.g., *BocFUL-BolK_2g60540*, *BocAP1-BolK_6g36610*, *BocAP3-1-BolK_4g38090*, *BocAP3-2-BolK_8g38150* and *BocPI-BolK_9g53730*) showed significant upregulation during the CB stage. Conversely, 13 DEGs incorporating known flowering repressors and inflorescence development regulators (e.g., *BocAGL24-1-BolK_1g20120*, *BocAGL6-BolK_4g07370*, *BocSVP-BolK_4g51100* and *BocAGL8-BolK_7g44950*) showed increased expression at the AIM stage (Figure 4D). Additionally, the C2H2-ZF transcription factor *BolK_2g02720*, TCP family members *BolK_4g19090* and *BolK_7g50000*, and the bZIP transcription factor *BolK_5g04900* were all significantly upregulated at the CB stage. In contrast, all WRKY family transcription factors (e.g., *BolK_4g58060*, *BolK_2g02920*, and *BolK_4g65390*) and several AP2 family members (*BolK_8g33960*, *BolK_4g2208*, and *BolK_7g42860*) were predominantly upregulated at the CM stage (Table S5).

### 2.4. DEGs Related to curd_vs_peduncle

**Figure 5:**
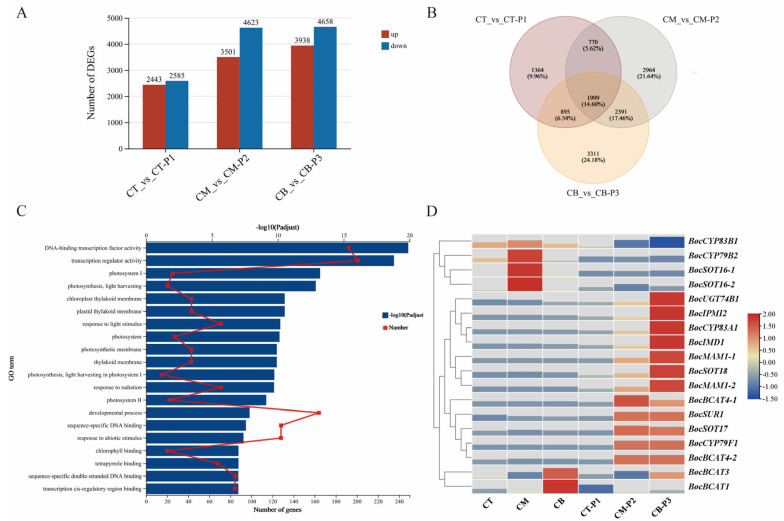
DEGs identified in three pairwise comparisons (CT_vs_CT-P1, CM_vs_CM-P2 and CB_vs_CB-P3). (**A**) Number of expressed genes between curd and peduncle. (**B**) Venn diagram of DEGs commonly or specifically expressed between curd and peduncle. (**C**) GO functional enrichment of DEGs between curd and peduncle. (**D**) Glucosinolate biosynthesis-related DEGs: curd vs. peduncle.

Analysis of DEGs in three groups of CT_vs_CT-P1, CM_vs_CM-P2 and CB_vs_CB-P3 were performed to identify the candidate genes regulating the developmental differences between AIM and the corresponding peduncle. For the comparisons of CT_vs_CT-P1, CM_vs_CM-P2 and CB_vs_CB-P3, we identified 5028, 8124, and 8596 differentially regulated genes, respectively (Figure 5A).

A total of 1999 overlapping DEGs were consistently shared across all three developmental stages in both AIM and peduncle, suggesting their potential key regulatory roles in coordinating the developmental relationship between these two interconnected tissues (Figure 5B). GO terms targeted by these overlapping DEGs were focused on the transcriptional activity regulation (GO:0003700 and GO:0140110) and developmental process (GO:0032502) (Figure 5C). Moreover, the KEGG analysis indicated that the pathways of photosynthesis antenna proteins (map00196) and plant hormone signal transduction (map04075), especially the brassinosteroid biosynthesis (map00905), were significantly enriched. The brassinosteroid biosynthesis gene *BolK_1g50530* showed upregulated expression in the peduncle, while *BolK_2g55630* and *BolK_8g34350* exhibited elevated expression in AIM. Interestingly, some important metabolic synthesis pathways were also specifically enriched, such as glucosinolate biosynthesis (map00966) and fatty acid elongation (map00062) (Figure S3).

In the glucosinolate (GSL) biosynthesis pathway, we identified 18 DEGs, among which 12 were significantly upregulated in the peduncle, and 6 were significantly upregulated in the AIM. Notably, glycosyltransferase gene *BocUGT74B1* (*BolK_5g21990*), sulfotransferase genes *BocSOT17* (*BolK_5g15450*) and *BocSOT18* (*BolK_6g47180*), cytochrome P450 gene *BocCYP83A1* (*BolK_4g41640*) were significantly upregulated in the peduncle at the CM-P2 and CB-P3 stages. Additionally, we observed elevated expression of two aminotransferase genes *BocBCAT4-2* (*BolK_5g43850*) and *BocSUR1* (*BolK_7g11460*) in the peduncle, which may be involved in the biosynthesis and hydrolysis of sinigrin, a GSL compound associated with bitterness and potential toxicity (Figure 5D and Table S6). The regulatory mechanisms underlying sinigrin metabolism merit further investigation.

### 2.5. DEGs Related to Peduncle Development

**Figure 6:**
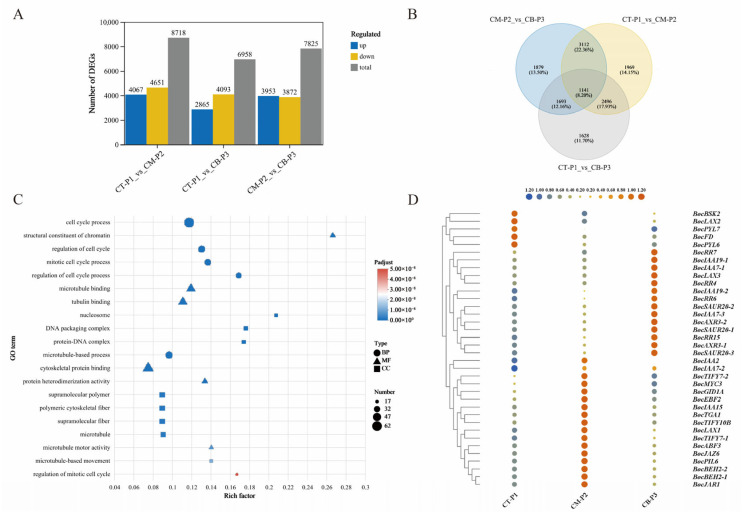
DEGs identified in three pairwise comparisons (CT-P1_vs_CM-P2, CT-P1_vs_CB-P3 and CM-P2_vs_CB-P3). (**A**) Number of expressed CT-P1_vs_CM-P2, CT-P1_vs_CB-P3 and CM-P2_vs_CB-P3. (**B**) Venn diagram of DEGs commonly or specifically expressedamong three stages of peduncle development. (**C**) GO functional enrichment of DEGs amongthree stages of peduncle development. (**D**) Phytohormone signaling DEGs: CT-P1_vs_CM-P2, CT-P1_vs_CB-P3 and CM-P2_vs_CB-P3.

To identify genes involved in peduncle development, we analyzed DEGs in the comparisons of CT-P1 vs. CM-P2, CT-P1 vs. CB-P3, and CM-P2 vs. CB-P3. A total of 8718, 6958 and 7825 genes were found to be differentially regulated for the above three comparisons, respectively (Figure 6A). Among these, 1141 DEGs, constituting 8.20% of the total detected genes, were co-expressed in the three comparison groups (Figure 6B). Subsequently, the possible genetic regulatory networks and pathways enriched by these 1141 overlapping DEGs were explored by GO and KEGG enrichment analysis.

Functional enrichment analysis revealed that the overlapping DEGs were predominantly associated with cytoskeletal organization and cell cycle regulation, as evidenced by significant enrichment in key GO terms: cell cycle process (GO:0022402), mitotic cell cycle process (GO:1903047), microtubule (GO:0008017) and cytoskeletal protein binding (GO:0008092) (Figure 6C). KEGG pathway analysis further supported these findings, showing significant enrichment in cell cycle-related pathways such as DNA replication and homologous recombination. Notably, we also observed marked enrichment in plant hormone signal transduction (particularly zeatin biosynthesis, map00908), carotenoid biosynthesis (map00906), and photosynthesis pathways (map00195) (Figure S4). These collective results suggest that the identified DEGs may coordinate cell division with hormonal regulation and metabolic processes to promote rapid peduncle elongation.

A total of 36 hormone-related DEGs were identified during peduncle development. These included AUX/IAA genes *BocIAA7-1* (*BolK_3g52380*), *BocIAA7-2* (*BolK_5g38920*), *BocIAA7-3* (*BolK_1g40960*), *BocIAA19-2* (*BolK_5g49700*) and response regulator genes *BocRR4* (*BolK_5g07030*), *BocRR6* (*BolK_3g62450*), *BocRR7* (*BolK_8g27790*), *BocRR15* (*BolK_6g32650*), which were significantly upregulated in the late stage of peduncle development (CB-P3) (Figure 6D and Table S7). Notably, *BolK_2g12030*, *BolK_7g15180*, *BolK_4g11940* and *BolK_2g35380* genes involved in zeatin metabolism exhibited marked upregulation during the peduncle development. These genes are functionally required for proper peduncle development and elongation (Table S7).

### 2.6. Validation of Curd and Peduncle Development-Related DEGs by qRT-PCR Analysis

**Figure 7:**
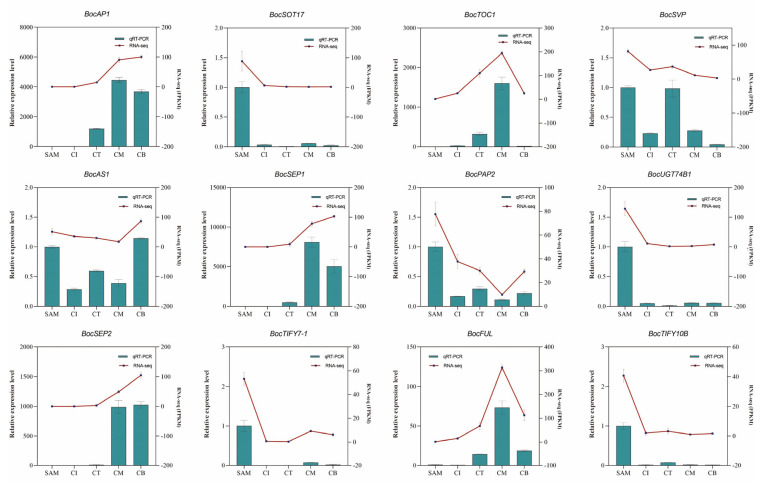
Validation of transcriptional expression levels of the representative DEGs by qRT-PCR. qRT-PCR was conducted to verify the expression levels of representative DEGs, with bar graphs displaying the mean values ± standard deviation.

To validate the reliability of our transcriptome data and further characterize key molecular events during curd development, we performed quantitative real-time polymerase chain reaction (qRT-PCR) analysis on 12 functionally representative genes selected from DEGs. These candidate genes were chosen based on their potential involvement in crucial biological processes such as phytohormone signaling (*BocTIFY7-1*, *BocTIFY10B*), floral transition and organ development (*BocSVP*, *BocFUL*, *BocLFY* and *BocAP1*), MADS-box gene regulation (*BocSEP1*, *BocSEP2*), glucosinolate metabolism (*BocSOT17*, *BocUGT74B1*), and circadian rhythm control (*BocTOC1*) (Figure 7). The qRT-PCR primer sequences are detailed in Table S8. Notably, the expression patterns of all selected genes showed strong concordance between qRT-PCR and RNA-seq data (correlation coefficient r > 0.85) (Figure S5), with consistent up- or downregulation trends across different developmental stages. This high degree of correlation not only confirmed the accuracy of our transcriptomic profiling but also reinforced the biological relevance of the identified DEGs in curd development. Furthermore, the successful validation of genes from diverse functional categories suggests the comprehensive nature of our transcriptome dataset in capturing key regulatory networks governing curd formation and development.

### 2.7. Subcellular Localization, Protein Structure and Regulatory Network Analysis of BocAP1, BocFUL, and BocSEP2

**Figure 8:**
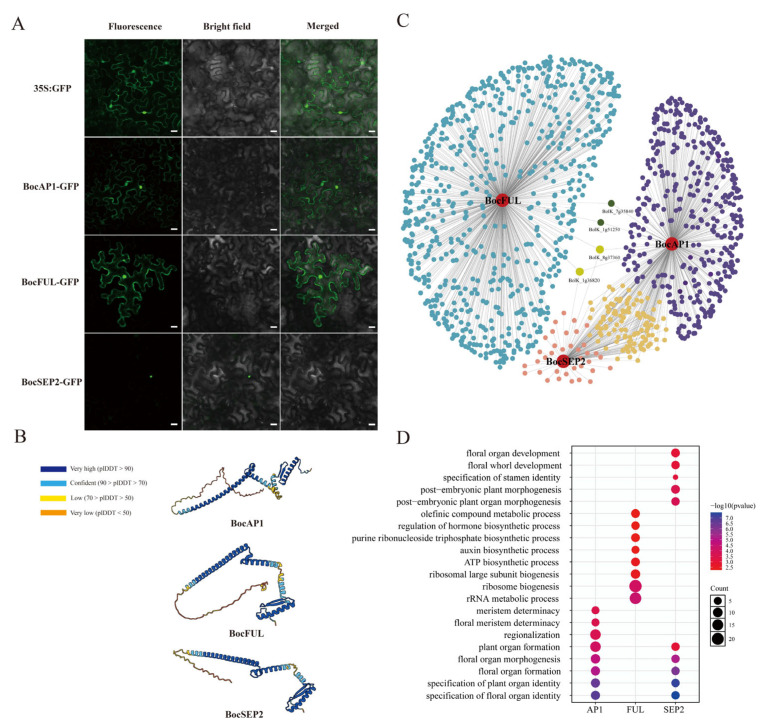
Multilevel functional and regulatory characterization of candidate genes. (**A**) Subcellular localization analysis. Scale bars, 20 µm. (**B**) Protein structure prediction. (**C**) Transcriptional regulatory network construction. Blue dots represent genes regulated by *BocFUL*, purple dots represent genes regulated by *BocAP1*, and orange dots represent genes regulated by *BocSEP2*. Other colors indicate genes that are co-regulated by two or all three transcription factors. (**D**) GO enrichment analysis.

We selected three key MADS-box transcription factors (*BocAP1*, *BocFUL* and *BocSEP2*) that are specifically upregulated during the late stage of curd development for further regulatory network analysis. Subcellular localization analysis indicated that *BocAP1* and *BocFUL* are localized to both the nucleus and the plasma membrane, whereas *BocSEP2* is localized to the nucleus, further supporting their roles as transcriptional regulators (Figure 8A). Protein structure predictions indicated that *BocAP1*, *BocFUL*, and *BocSEP2* are predominantly composed of α-helices, with notable differences in their overall three-dimensional conformations, suggesting potential functional divergence among these MADS-box proteins (Figure 8B).

Joint analysis of the regulatory networks of these transcription factors identified a batch of potential associated genes. Among them, *BocFUL* regulates the largest number of potential downstream genes, followed by *BocAP1*, and then *BocSEP2*. Interestingly, there are certain genes that are co-regulated by these three transcription factors. The greatest overlap is observed between *BocAP1* and *BocSEP2*, which share 114 co-regulated genes, whereas *BocAP1* and *BocFUL* share only 2. Moreover, genes *BolK_1g51250* and *BolK_7g35840* are co-regulated by all three transcription factors (Figure 8C).

GO enrichment analysis of genes in the regulatory network revealed that the differentially expressed genes were mainly enriched in biological processes closely related to flower development, including floral organ formation, perianth whorl development, and stamen-specific determination. In addition, significant enrichment was observed in hormone biosynthesis and regulation, as well as ATP- and ribosome-related metabolic and biosynthetic processes, suggesting that these genes may not only participate in floral organ formation and organ identity determination, but also potentially regulate flower organogenesis through energy metabolism and protein synthesis pathways (Figure 8D).

## 3. Discussion

**Figure 9:**
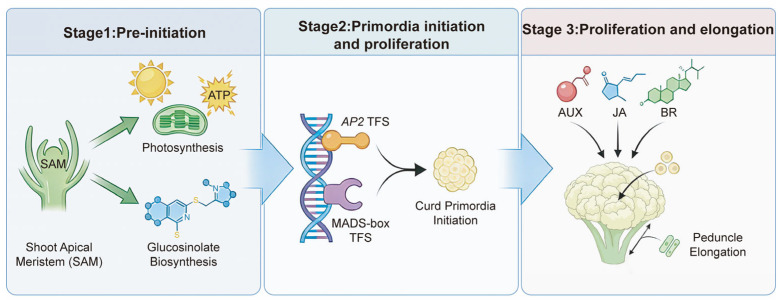
Schematic diagram illustrating key regulatory factors at the SAM (stage 1), curd proliferation (stage 2) and peduncle elongation (stage 3).

The differentiation and development of the curd represent one of the most critical stages in cauliflower growth, as curd formation not only determines the final commercial traits but is also closely linked to the plant’s reproductive development strategy. Previous studies have shown that the curd phenotype of cauliflower resembles that of the *Arabidopsis ap1*/*cal* double mutant, both exhibiting a characteristic structure of incompletely differentiated floral organs arranged in a compact pattern [6]. Moreover, a premature stop codon has been identified in the fifth exon of the *BobCAL* gene in cauliflower, and its functional loss is hypothesized to underlie the genetic basis of curd formation [5]. However, despite the identification of a few candidate genes, the genetic basis and regulatory network underlying curd development in cauliflower remain largely uncharacterized. In recent years, RNA-seq has been widely applied to investigate gene expression during plant growth and development, providing a powerful tool for constructing regulatory maps of complex traits [17]. In this study, we employed high-throughput transcriptome sequencing using cauliflower inbred line ZAASC4101, collecting curd and peduncle tissues at different developmental stages; the CI, CT, and CM stages correspond to the AIM state, during which the meristem proliferates without acquiring floral identity, whereas the CB stage represents the transition to the FM stage. The aim was to identify key regulatory genes involved in curd differentiation and floral transition, and to explore potential regulatory networks. These results provide a foundation for elucidating the molecular mechanisms underlying curd development in cauliflower. In addition to photoperiod and temperature, environmental factors such as nutrient availability, water status, light quality, and abiotic stresses are known to modulate the vegetative-to-reproductive transition in plants and may influence curd development in cauliflower. We propose a stage-specific regulatory model in which metabolic priming at the SAM stage, transcription factor–driven meristem proliferation during curd expansion, and phytohormone-mediated elongation at the bolting stage collectively coordinate curd development (Figure 9). Our study in cauliflower shares key similarities with broccoli curd transcriptomics: in both cases, SAM and early curd stages involve activation of *AP2*/MADS-box transcription factors, hormone signaling pathways (e.g., AUX, GA/JA), and enhanced photosynthetic or metabolic activity, highlighting conserved regulatory networks underlying curd formation in *Brassica* crops [18].

During the transition of cauliflower from the vegetative to the reproductive growth stage, there is a marked remodeling of organ structure and function [19]. The SAM, as a typical vegetative organ, is morphologically green, rich in chlorophyll, and possesses strong photosynthetic capacity [20]. In contrast, as the curd begins to differentiate, its tissue color gradually shifts to near white, with a significant decline in chlorophyll content, indicating weakened photosynthetic activity and a functional shift toward storage and protective structures. Chlorophyll a/b binding proteins and PSI/PSII subunits (PsaG/K, PsbW/X/Y/27) constitute the core light-harvesting complexes [21], and in our transcriptome analysis, these photosynthesis-related genes were significantly upregulated in the SAM. Representative genes (e.g., *BolK_3g34500*, *BolK_7g17030*, *BolK_8g53480*) were highly expressed, consistent with the SAM’s strong photosynthetic activity. In addition, glucosinolate (GSL) metabolism is a specialized secondary metabolic pathway unique to *Brassicaceae* species [22]. Glucosinolate biosynthesis partially relies on chloroplast-derived substrates and enzymatic conditions, leading to enhanced expression in photosynthetically active, chloroplast-rich tissues [23,24]. Key glucosinolate biosynthetic genes—including glycosyltransferase (*BolK_5g21990*), sulfotransferases (*BolK_2g31760*, *BolK_5g15450*, *BolK_6g47200*), and cytochrome P450s (*BolK_1g01200*, *BolK_4g41640*)—were markedly upregulated in the SAM versus curd, and also higher in the peduncle with light green in color, suggesting that aliphatic GSL synthesis is more active in photosynthetic tissues during the vegetative–reproductive transition.

Curd development is fundamentally a complex process characterized by sustained proliferation of the AIM and suppression of floral differentiation. This process is regulated not only by canonical flowering genes such as *CAL* and *AP1*, but may also involve previously uncharacterized regulatory networks [6]. In this study, we identified a set of genes specifically upregulated during the CI, CT, and CB stages. Several of these genes, including *BolK_2g56440*, *BolK_7g39790*, and *BolK_8g33800*, belong to the *CYCLIN* family and are known in *Arabidopsis* to control rapid cell proliferation through phosphorylation cascades mediated by *CYCLIN*–*CDK* complexes, reflecting a highly regulated spatiotemporal mechanism underlying plant indeterminate growth [25,26]. In addition, we identified novel candidate genes potentially involved in curd development. These may represent functional innovations derived from the *Brassica*-specific whole-genome triplication, where sub- or neo-functionalization has conferred new roles in meristem regulation. For example, *BolK_9g53110* (sucrose synthase) may be orthologous to *AtSUS4*, a known downstream target of *AtIDD8* in *Arabidopsis* that integrates photoperiod and sugar signaling to promote floral transition [27]. Other candidates, such as *BolK_1g01560* (calcium-dependent lipid-binding protein), *BolK_1g20390* (pectin lyase-like protein), and *BolK_4g43910* (phosphoenolpyruvate carboxykinase), may also play essential roles in coordinating metabolic and structural cues during curd morphogenesis.

The transition of cauliflower from the AIM to the flowering stage is a critical step in its reproductive development. This process involves the reprogramming of meristem identity and the activation of floral organ development pathways. During this transition, the AIM gradually loses its indeterminate growth capacity and transforms into a determinate FM, initiating the formation of specific floral organs [28]. This developmental switch is jointly regulated by environmental cues (such as photoperiod and temperature) and endogenous signals (such as hormones and sugars) [29,30]. At the molecular level, it is driven by the activation of multiple floral integrator genes, which integrate upstream signals and activate key downstream transcription factors that coordinate the floral transition network [31]. Among the downstream regulators, *AP2*-like and MADS-box transcription factors play central roles. *AP2* (*APETALA2*), a member of the *AP2*/ERF transcription factor family, regulates floral organ boundaries, phyllotaxy, and early floral patterning [32]. In this study, we found that *AP2* family members (*BolK_8g33960*, *BolK_4g22080*, and *BolK_7g42860*) were predominantly upregulated at the CT stage. MADS-box transcription factors constitute the core regulators that specify floral organ identities [33]. Among them, the *SEPALLATA* (*SEP1*–*SEP4*) genes, which encode E-class MADS-box proteins, are essential for proper floral organ development [34]. *SEP* genes exhibited marked upregulation during the late stages of curd development, notably *BolK_3g09030*, *BolK_1g58900*, *BolK_3g72920*, and *BolK_2g51870*. Overall, the transition from AIM to flowering in cauliflower is governed by a hierarchical transcriptional regulatory network, in which MADS-box and *AP2*-like transcription factors play central roles. The transcription factor regulatory network inferred here, based on motif presence and expression correlation, represents candidate rather than confirmed regulatory relationships. While qRT-PCR supports the reliability of the transcriptomic data, functional genetic validation, such as mutant or overexpression lines, is lacking, particularly for key regulators such as *BocAP1*, *BocFUL*, and *BocSEP2*. Future studies using alternative computational methods, including GENIE3 and WGCNA, or experimental approaches, including DAP-seq and ChIP-seq, will be valuable to validate and refine these predicted interactions.

Plant hormones play crucial roles in regulating cauliflower development, especially during rapid peduncle elongation [35,36]. In peduncle tissues, key auxin-related genes—including AUX/IAA repressors (*BolK_1g10580*, *BolK_3g52380*, *BolK_5g49700*), auxin-inducible factors (*BolK_2g09120*, *BolK_2g09150*, *BolK_3g10470*), and GH3 enzymes (*BolK_4g01820*)—were significantly upregulated, forming a core auxin signaling module regulating cell elongation and tissue polarity. TIFY family genes (*BolK_2g28270*, *BolK_2g30100*, *BolK_6g32600*) and bHLH-MYC domain proteins (e.g., *BolK_7g30730*) involved in jasmonic acid signaling were also upregulated, supporting JA’s role in peduncle growth as shown in *Arabidopsis* [37]. Brassinosteroid signaling components, including BES1_N domain transcription factors (*BolK_7g61850*, *BolK_1g02630*), were elevated, indicating BR’s function in promoting stem elongation via cell wall remodeling. Previous studies confirmed that BZR1/BES1 interact with auxin pathways to regulate cell elongation genes [38], a network likely conserved in cauliflower. Overall, auxin, jasmonic acid, and brassinosteroids coordinate cell expansion and structural development in the peduncle, offering molecular targets for improving curd morphology and plant architecture.

## 4. Materials and Methods

### 4.1. Experimental Material

**Figure 10:**
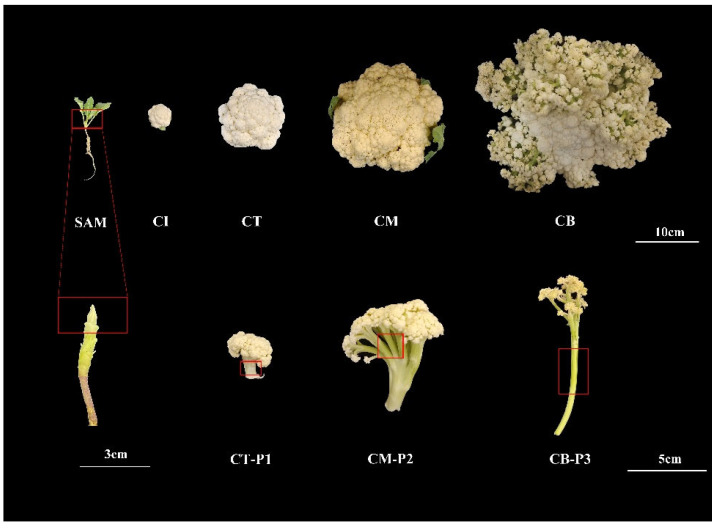
Phenotypes of cauliflower curd and peduncle development. SAM samples were taken from 25-day-old seedlings at the four-true-leaf stage. Curd samples were collected at four stages (CI: curd initiation stage; CT: curd thickening stage; CM: curd maturation stage; CB: curd bolting stage), and peduncle samples at three corresponding stages (CT-P1: peduncle at expansion stage; CM-P2: peduncle at maturation stage; CB-P3: peduncle at elongation stage).

The F7 generation cauliflower inbred line ZAASC4101, derived from a commercial variety of YinXue70 (Wuhan Seed Industry Co., Ltd., Wuhan, China) through multiple generations of self-pollination, was used as the plant material. Seeds were sown on 20 July 2023 and seedlings were transplanted into pots with commercial matrix on 20 August 2023, followed by growth in an artificial climate chamber (23 °C/20 °C day/night; 16 h light/8 h dark). Three distinct tissues—SAM, curd, and peduncle—were sampled from ZAASC4101 at different developmental stages and used for subsequent analyses (Figure 10).

### 4.2. Library Construction and Sequencing

Total RNA was extracted from three biological replicates per group using QIAzol Lysis Reagent (Qiagen, Hilden, Germany) according to the manufacturer’s instructions. To eliminate genomic DNA contamination, RNA samples were treated with DNase I (Takara, Bio, Shiga, Japan). RNA integrity and quality were evaluated using the Agilent 5300 Bioanalyzer (Agilent Technologies, Santa Clara, CA, USA), and RNA concentration was measured with a NanoDrop ND-2000 spectrophotometer (NanoDrop Technologies, Wilmington, DE, USA). A total of 24 RNA samples exhibiting high quality—characterized by OD260/280 ratios between 1.8 and 2.2, OD260/230 ratios ≥ 2.0, RNA Quality Number (RQN) ≥ 6.5, 28S/18S rRNA ratios ≥ 1.0, and RNA amounts exceeding 1 μg—were selected for downstream library preparation.

The RNA-seq transcriptome libraries for SAM, curd, and peduncle were prepared using the Illumina^®^ Stranded mRNA Prep, Ligation kit (Illumina, San Diego, CA, USA) following the manufacturer’s protocol, starting with 1 μg of total RNA. Firstly, mRNA was isolated from total RNA by magnetic beads with Oligo (dT) that specifically bind to the poly-A tail, followed by fragmentation in fragmentation buffer. Secondly, double-stranded cDNA was synthesized using the SuperScript double-stranded cDNA synthesis kit (Invitrogen, Carlsbad, CA, USA) with random hexamer primers. The cDNA then underwent end-repair, phosphorylation, and adapter ligation according to the library construction instructions. Libraries were size-selected for cDNA fragments of approximately 300 bp using 2% Low Range Ultra Agarose gel electrophoresis and then PCR-amplified for 15 cycles with Phusion DNA polymerase (NEB, Beijing, China). The final libraries were quantified using Qubit 4.0 fluorometer (Thermo Fisher, San Diego, CA, USA) and sequenced on the NovaSeq X Plus platform (Illumina, San Diego, CA, USA) with paired-end 150 bp reads (PE150) using the NovaSeq Reagent Kit.

### 4.3. Quality Control and Read Mapping

Clean data were obtained by trimming and quality filtering of raw paired-end reads using fastp v0.23.4 software [39] to ensure high-quality downstream analysis. The clean reads were then individually aligned to the reference genome using HISAT2 [40], followed by evaluation of alignment quality. Transcriptome assembly for each sample was performed in a reference-guided manner using StringTie [41].

### 4.4. RNA-Seq Data Analysis

Quantitative analysis of gene and transcript expression levels was performed using RSEM v1.3.1 software (http://deweylab.github.io/RSEM/ (accessed on 20 January 2024)). After obtaining gene read counts, differential expression analysis between two samples was conducted using DESeq2 v1.34.0 (http://bioconductor.org/packages/stats/bioc/DESeq2/ (accessed on 20 January 2024)). Genes with a false discovery rate (FDR) less than 0.05 and an absolute log2 fold change (|log_2_FC|) equal to or greater than 1 were considered significantly differentially expressed.

GO functional enrichment and KEGG pathway analyses were performed using GOATOOLS v1.4.12 (https://github.com/tanghaibao/GOatools (accessed on 20 January 2024)) and Python SciPy library (https://scipy.org/install/ (accessed on 20 January 2024)), respectively. DEGs from each comparison group were subjected to enrichment analysis against the GO database (http://geneontology.org/ (accessed on 20 January 2024)) and the KEGG pathway database (https://www.genome.jp/kegg/ (accessed on 20 January 2024)). Significantly enriched GO terms and metabolic pathways were identified using a Bonferroni-corrected *p*-value threshold of <0.05.

Cluster number was initially guided by prior time-series studies in the field (typically nine clusters) and clustering was performed using the Mfuzz R package (version 2.66.0). The optimal number of clusters was evaluated using Dmin, Partition Coefficient, Partition Entropy, and average silhouette width. Clusters were retained only if they displayed coherent temporal expression patterns and most genes had high membership values (>0.7) in a single cluster.

### 4.5. Real-Time Quantitative PCR Analysis

qRT-PCR was performed to validate the expression of DEGs. Twelve DEGs were randomly selected for analysis. Total RNA was extracted from SAM, curd, and peduncle samples, and reverse transcribed into cDNA using the cDNA Synthesis SuperMix (TransGen Biotech, Beijing, China). Fluorescence measurements were carried out according to the kit protocol (Vazyme, Nanjing, China). Each biological replicate was analyzed with three technical replicates. Relative expression levels were calculated using the 2^−∆∆Ct^ method [42], with *ACT2* serving as the reference gene. Primer sequences are listed in Table S8.

### 4.6. Subcellular Localization and Protein Structure Prediction

A combination of subcellular localization and protein structure prediction was employed to investigate the functional characteristics of the *BocAP1*, *BocFUL* and *BocSEP2* proteins. First, the coding sequence of the target genes, with the stop codon removed, was cloned into the pCGFP vector at the *BamHI* and *XhoI* restriction sites under the control of the constitutive CaMV 35S promoter to generate a GFP fusion construct (primer sequences are listed in Table S9). The recombinant plasmid and the empty vector control were introduced into Agrobacterium tumefaciens strain GV3101 via the freeze–thaw method and used to infiltrate the leaves of four-week-old tobacco plants. After 48 h of dark incubation, GFP fluorescence signals were detected and recorded using an Olympus confocal laser scanning microscope. Simultaneously, the three-dimensional structure of the target protein was predicted using the AlphaFold online server (https://alphafoldserver.com/ (accessed on 1 August 2024)) to support functional analysis.

### 4.7. Prediction of Transcription Factor Target Genes and Construction of Regulatory Networks

To identify potential target genes of key transcription factors (TFs), homologous proteins in *Arabidopsis thaliana* were first retrieved via BLASTP. The binding motif sequences of these homologous TFs were obtained from JASPAR databases and saved in MEME format. Using the *Brassica oleracea* Korso reference genome, the 2000 bp upstream regions from the transcription start site of each gene were extracted as promoter sequences with BEDTools. Potential TF binding sites were then scanned in the promoter sequences using FIMO from the MEME Suite v5.4.1 (https://meme-suite.org/meme/ (accessed on 20 August 2025)), with the MEME motif files of the *Arabidopsis* homologs as input. Candidate binding sites were identified based on a significance threshold of *p* < 1 × 10^−4^. To increase confidence, RNA-seq expression data were integrated to calculate the Pearson correlation coefficients between TFs and candidate target genes. Gene pairs with |r| ≥ 0.9 and *p* < 0.05 were considered significantly correlated. Only genes supported by both motif-based prediction and expression correlation were retained as high-confidence candidate targets. Finally, the regulatory network was constructed and visualized using Cytoscape 3.10.2 and GO enrichment analysis of genes in the regulatory network.

## 5. Conclusions

Transcriptome profiling of cauliflower curd and peduncle across developmental stages uncovered critical regulatory networks governing curd formation. Our analysis revealed high expression of photosynthesis and glucosinolate biosynthesis genes in the SAM, indicating their importance in early metabolic activity. During SAM proliferation and peduncle elongation, we observed pronounced activation of auxin, jasmonic acid, and brassinosteroid signaling pathways, underscoring their pivotal roles in structural development. Notably, the floral transition and meristem patterning were marked by the upregulation of key transcription factors, particularly MADS-box family members. These findings provide molecular-level insights into curd morphogenesis and identify promising targets for molecular breeding strategies aimed at improving cauliflower quality traits.

## Figures and Tables

**Table 1:** Overview of the transcriptome sequencing data.

Sample	Raw Reads (M)	Clean Reads (M)	Clean Bases (Gb)	Q20 (%)	Q30 (%)	Clean Reads Ratio (%)	Total Mapped (%)	Uniquely Mapped (%)
SAM_1	44.76	43.90	65.61	96.80	90.86	98.08	91.44	88.69
SAM_2	45.08	44.25	66.00	97.01	91.37	98.14	91.68	89.00
SAM_3	44.79	43.87	65.47	96.79	90.78	97.95	91.35	88.73
CI_1	124.27	121.51	181.68	96.81	90.79	97.78	91.69	88.77
CI_2	47.92	46.91	70.17	96.32	89.56	97.90	90.85	88.21
CI_3	47.90	46.89	70.07	96.46	89.97	97.88	91.33	88.72
CT_1	41.51	40.66	60.71	96.94	91.12	97.95	91.73	89.05
CT_2	45.70	44.85	66.93	96.83	90.86	98.13	91.60	88.92
CT_3	43.46	42.59	63.47	96.95	91.17	98.00	91.83	89.14
CM_1	42.11	41.25	61.56	96.95	91.15	97.96	92.07	89.31
CM_2	45.57	44.71	66.73	96.87	90.98	98.12	91.92	89.16
CM_3	45.19	44.39	66.21	96.90	91.05	98.23	91.81	89.08
CB_1	41.34	40.53	60.56	96.96	91.17	98.05	91.50	88.67
CB_2	44.96	43.76	64.66	96.95	91.18	97.33	92.08	89.30
CB_3	45.70	44.49	65.80	97.02	91.34	97.36	92.03	89.20
CT-P1_1	47.35	46.10	68.16	97.03	91.37	97.37	92.12	89.50
CT-P1_2	44.88	43.84	64.45	97.12	91.60	97.67	92.24	89.68
CT-P1_3	47.48	46.03	67.47	97.13	91.67	96.95	92.21	89.85
CM-P2_1	48.34	46.60	68.26	97.12	91.65	96.41	91.93	89.34
CM-P2_2	50.62	49.21	72.28	97.18	91.77	97.21	92.28	89.73
CM-P2_3	42.03	41.01	60.88	97.12	91.59	97.58	92.60	90.00
CB-P3_1	52.24	50.66	74.66	97.20	91.82	96.98	92.56	90.00
CB-P3_2	49.87	48.61	71.15	97.20	91.81	97.48	92.84	90.41
CB-P3_3	50.65	49.36	72.26	97.21	91.84	97.46	92.49	90.05

## Data Availability

The sequencing data presented in this study are openly available in the Genome Sequence Archive (GSA) database at the National Genomics Data Center (NGDC), China National Center for Bioinformation (https://ngdc.cncb.ac.cn/gsa (accessed on 10 October 2025)) under the accession number CRA031042, accessed on 9 October 2027.

## Supplementary Materials

The following supporting information can be downloaded at: https://www.mdpi.com/article/10.3390/ijms27031308/s1.
