# Supplementary material for: Dynamic Transcriptome Profiling Reveals Key Regulatory Networks Underlying Curd Development in Cauliflower (Brassica oleracea L. botrytis)
Source: Int J Mol Sci. 2026 Jan 28;27(3):1308. doi: 10.3390/ijms27031308 (PMC12897975; doi:10.3390/ijms27031308)
Supplement: Supplementary file 1 [file ijms-27-01308-s001.zip › Supplementary Fig.pdf]

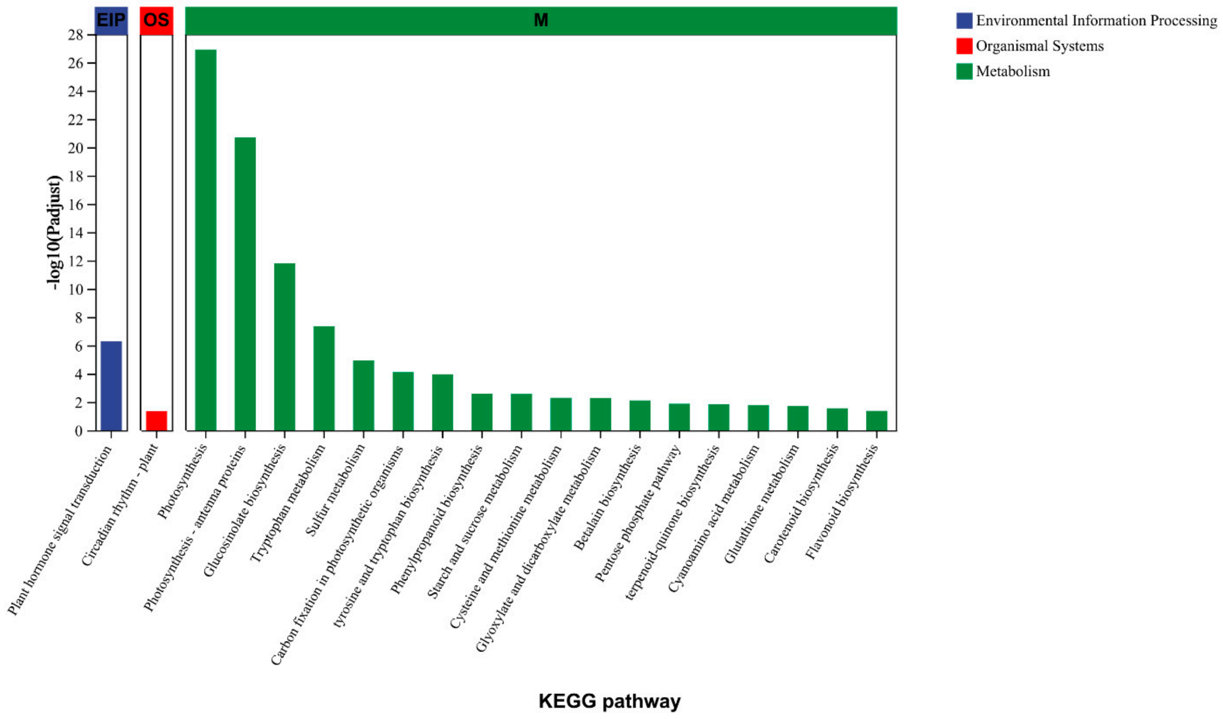

Figure S1 KEGG enrichment of DEGs between SAM and three stages of curd development.

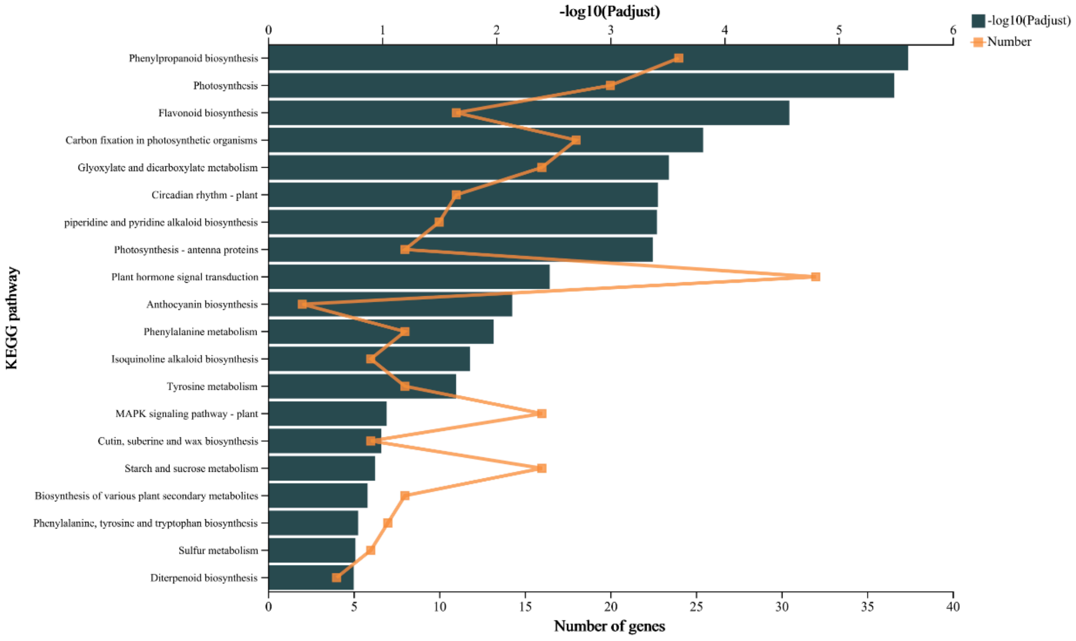

Figure S2 KEGG enrichment of DEGs between CB and three stages of curd development.

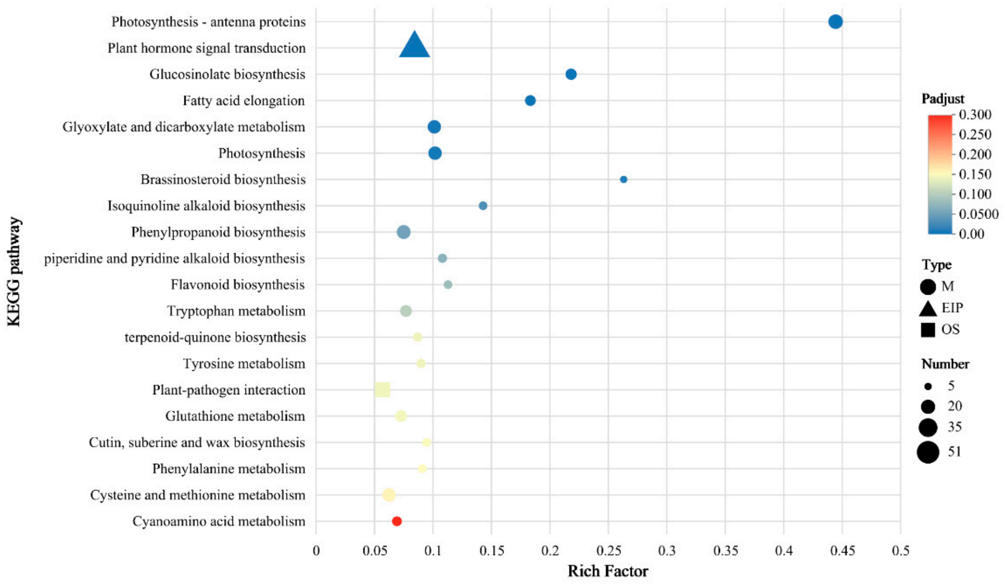

Figure S3 KEGG enrichment of DEGs between curd and peduncle.

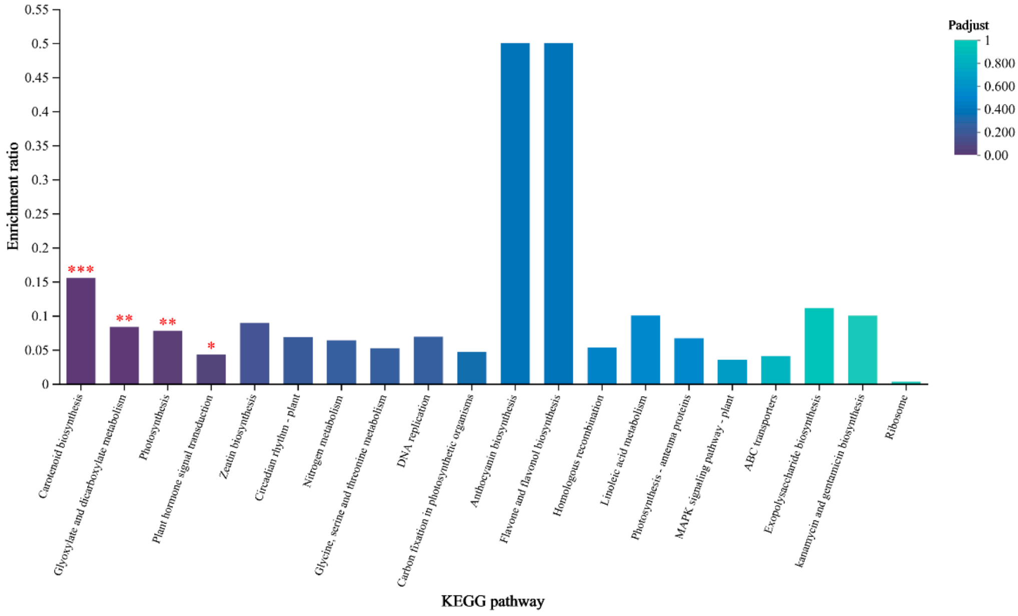

Figure S4 KEGG enrichment of DEGs related to peduncle development.

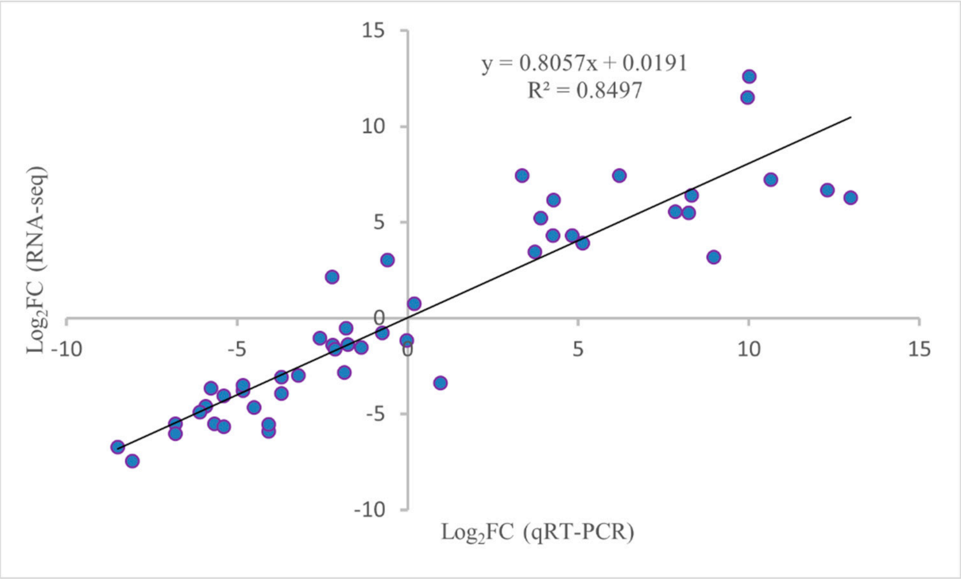

Figure S5 Correlations between RNA-seq and qRT-PCR gene expression data.
